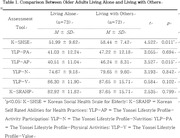# Comparative research on social health between older adults living alone and living with others

**DOI:** 10.1002/alz.085691

**Published:** 2025-01-09

**Authors:** Jung Min Han

**Affiliations:** ^1^ Yonsei university, Wonju‐si, Gangwon‐do Korea, Republic of (South)

## Abstract

**Background:**

The aim of this study is to find out differences in social health between older adults living alone and living with others.

**Method:**

A survey was conducted on 73 older adults who live alone and 72 older adults who live with others aged 60 or older. Living alone was surveyed by general information, social health was surveyed by Korean Social Health Scale for Elderly (K‐SHSE), and lifestyle was surveyed by The Yonsei Lifestyle Profile (YLP). Lifestyle values were surveyed by The Yonsei Lifestyle Profile‐Values (YLP‐V), and self‐esteem was surveyed by Korean Self Rated Abilities for Health Practices (K‐SRAHP). Independent sample *t*‐test was used to compare the differences between older adults living alone and living with others.

**Result:**

As a result of social health evaluation tool K‐SHSE showed a significant difference of 58.44±7.42 score for older adults living with others and 51.99±9.62 score for living alone (*p* = 0.011). Also, results of Activities Participation in YLP‐AP showed a significant difference of 46.24±8.31 score for older adults living with others and 40.51±11.04 score for living alone (*p* = 0.015).

**Conclusion:**

There was a significant difference between the social health levels between older adults living alone compared to older adults living with others. Therefore, it is expected that follow‐up studies will conduct intervention research that can improve the social health of older adults living alone.